# Interactions between HIV-1 Vif and human ElonginB-ElonginC are important for CBF-β binding to Vif

**DOI:** 10.1186/1742-4690-10-94

**Published:** 2013-08-29

**Authors:** Xiaodan Wang, Xiaoying Wang, Haihong Zhang, Mingyu Lv, Tao Zuo, Hui Wu, Jiawen Wang, Donglai Liu, Chu Wang, Jingyao Zhang, Xu Li, Jiaxin Wu, Bin Yu, Wei Kong, Xianghui Yu

**Affiliations:** 1 National Engineering Laboratory for AIDS Vaccine, College of Life Science, Jilin University, Changchun, Jilin Province, People’s Republic of China; 2 Key Laboratory for Molecular Enzymology and Engineering of the Ministry of Education, College of Life Science, Jilin University, Changchun, Jilin Province, People’s Republic of China

**Keywords:** CBF-β, Elongin BC complex, HIV-1, Protein binding, Ubiquitin-protein ligases, Vif

## Abstract

**Background:**

The HIV-1 accessory factor Vif is necessary for efficient viral infection in non-permissive cells. Vif antagonizes the antiviral activity of human cytidine deaminase APOBEC3 proteins that confer the non-permissive phenotype by tethering them (APOBEC3DE/3F/3G) to the Vif-CBF-β-ElonginB-ElonginC-Cullin5-Rbx (Vif-CBF-β-EloB-EloC-Cul5-Rbx) E3 complex to induce their proteasomal degradation. EloB and EloC were initially reported as positive regulatory subunits of the Elongin (SIII) complex. Thereafter, EloB and EloC were found to be components of Cul-E3 complexes, contributing to proteasomal degradation of specific substrates. CBF-β is a newly identified key regulator of Vif function, and more information is needed to further clarify its regulatory mechanism. Here, we comprehensively investigated the functions of EloB (together with EloC) in the Vif-CBF-β-Cul5 E3 ligase complex.

**Results:**

The results revealed that: (1) EloB (and EloC) positively affected the recruitment of CBF-β to Vif. Both knockdown of endogenous EloB and over-expression of its mutant with a 34-residue deletion in the COOH-terminal tail (EloBΔC34/EBΔC34) impaired the Vif-CBF-β interaction. (2) Introduction of both the Vif SLQ → AAA mutant (VifΔSLQ, which dramatically impairs Vif-EloB-EloC binding) and the Vif PPL → AAA mutant (VifΔPPL, which is thought to reduce Vif-EloB binding) could reduce CBF-β binding. (3) EloB-EloC but not CBF-β could greatly enhance the folding of full-length Vif in *Escherichia coli*. (4) The over-expression of EloB or the N-terminal ubiquitin-like (UbL) domain of EloB could significantly improve the stability of Vif/VifΔSLQ/VifΔPPL through the region between residues 9 and 14.

**Conclusion:**

Our results indicate that the Vif interaction with EloB-EloC may contribute to recruitment of CBF-β to Vif, demonstrating that the EloB C-teminus may play a role in improving Vif function and that the over-expression of EloB results in Vif stabilization.

## Background

The human immunodeficiency virus type 1/acquired immunodeficiency syndrome (HIV-1/AIDS) epidemic is a global health concern. *Vif*, as an accessory gene of HIV-1, plays pivotal roles in both the early and late steps of the HIV-1 life cycle [1-3]. Vif is necessary for efficient viral replication in non-permissive cell lines [4], and APOBEC3 host proteins (APOBEC3B, APOBEC3DE, APOBEC3G and APOBEC3F) [5] are sufficient to confer the non-permissive phenotype [6-11]. They possess strong antiviral activities based on deamination of retroviral complementary DNA [12]. Vif counteracts the anti-HIV activity mainly by recruiting the APOBEC3 proteins (APOBEC3DE, APOBEC3G and APOBEC3F) [5], the transcription cofactor CBF-β and EloB-EloC to the Cul5-Rbx complex, thereby forming an E3 ubiquitin ligase and ultimately inducing the proteasomal degradation of these antiviral host proteins [10,13-17].

As the formation of the E3 ubiquitin ligase complex is a prerequisite for Vif to neutralize APOBEC3 proteins, the functional domains that Vif utilizes to recruit the E3 complex have been well studied [18,19]. The N-terminal region of Vif contains the main sites involved in binding of CBF-β and APOBEC3 proteins [17,20-25], while its C-terminal domain contains a so-called SOCS-box motif, which is responsible for binding to the EloB-EloC complex [26-28], and a conserved HCCH zinc coordination site that mediates selective binding to Cul5 [29-32]. Every component of the Vif-Cul5 E3 ligase is indispensable for degradation of APOBEC3 proteins. Vif acts as a substrate adaptor molecule to bridge APOBEC3G (A3G) with the Cul5 E3 ligase [13], and the newly found factor CBF-β is reported to be a unique regulator of Vif-Cul5 E3 ligase by promoting folding of Vif [25,33-36]. EloB and EloC are also known as regulatory subunits, whereas Cul5 functions as a scaffold protein [13].

EloB and EloC are two ubiquitous proteins that form a heterodimer complex. They were originally found in the Elongin (SIII) complex, which was proposed to contain three subunits: EloA, EloB and EloC [37,38]. EloA is known as a positive transcriptional elongation factor of RNA polymerase II, whereas EloB and EloC act as positive regulators of EloA [37,38]. Soon afterward, EloB and EloC were identified in many cullin-RING ubiquitin ligase complexes [13,39-42], forming the cullin E3 core together with cullin family proteins Cul2/Cul5 and a RING finger protein Rbx [43]. In these E3 ligase complexes, EloB-EloC binds to substrate-targeting subunits via a conserved motif called the BC-box [42-44] in order to mediate polyubiquitination and ultimately proteasomal degradation of the targeted substrates. These substrate-binding proteins, which are characterized by the SOCS-box motif containing the N-terminal BC-box sequence [42-44], typically include cellular proteins such as von Hippel-Lindau (VHL) tumor suppressor [40,45] and the suppressor of cytokine signaling (SOCS) [41,42,46], with corresponding substrates HIF-1α [40,45] and Janus kinases (JAKs) [41,42,46]. The substrate-binding proteins may also be viral proteins, such as adenovirus E4orf6 [39] and HIV-1 Vif [13], which target the substrates p53 [39] and the well-known APOBEC3 proteins [13], respectively.

EloB is a highly conserved cellular factor in humans, rats, *Drosophila melanogaster* and *Caenorhabditis elegans*[47]. As a member of the third class of ubiquitin family proteins, it is a 118-amino acid protein composed of an 84-amino acid N-terminal (ubiquitin-like) UbL domain and a 34-amino acid COOH-terminal tail, both of which are highly conserved [47,48]. The EloB UbL domain is necessary and sufficient for binding to EloC [47,48], whereas its C-terminal tail has been reported to interact with SOCS2 and SOCS4 proteins, significantly stabilizing the SOCS2 E3 complex and assisting folding of the SOCS4 protein [49,50]. In the Vif-CBF-β-Cul5 E3 complex, the C-terminus of EloB is known to bind to the PPLP motif of Vif, but without an ascribed function as yet [26].

In this study, we demonstrated that the interactions between Vif and EloB-EloC are important for CBF-β recruitment to Vif. Both knockdown of endogenous EloB (together with EloC) and over-expression of EloBΔC34, an EloB mutant with a 34-residue deletion in the COOH-terminal tail, impaired the Vif-CBF-β interaction and stabilized the A3G level. The interaction with EloB-EloC, but not CBF-β, was shown to greatly increase the solubility of full-length Vif in *Escherichia coli*, and in cells, both the introduction of the VifΔSLQ mutant, which dramatically impairs Vif-EloB-EloC binding, and that of the VifΔPPL mutant, which is considered to reduce Vif-EloB binding, were found to impair Vif-CBF-β binding [33,36]. Moreover, we showed that over-expression of EloB or EloBΔC34 could significantly prevent degradation of Vif/VifΔSLQ/VifΔPPL, indicating that the EloB N-terminal UbL domain may enhance the stability of Vif in an unknown manner. We also found that EloB stabilized Vif mainly through the same region (between residues 9 and 14) by which it interacted with EloC. Overall, our study is helpful in further understanding the interactions among components of the Vif-CBF-β-Cul5 E3 complex, demonstrates that the C-terminus of EloB, which is not required for EloC binding and degradation of P53, plays a role in improving Vif function and expands the diversity of functions of the UbL protein EloB.

## Results

### Knockdown of endogenous EloB expression impairs Vif-induced A3G degradation and decreases HIV-1 virion infectivity

**Figure 1:**
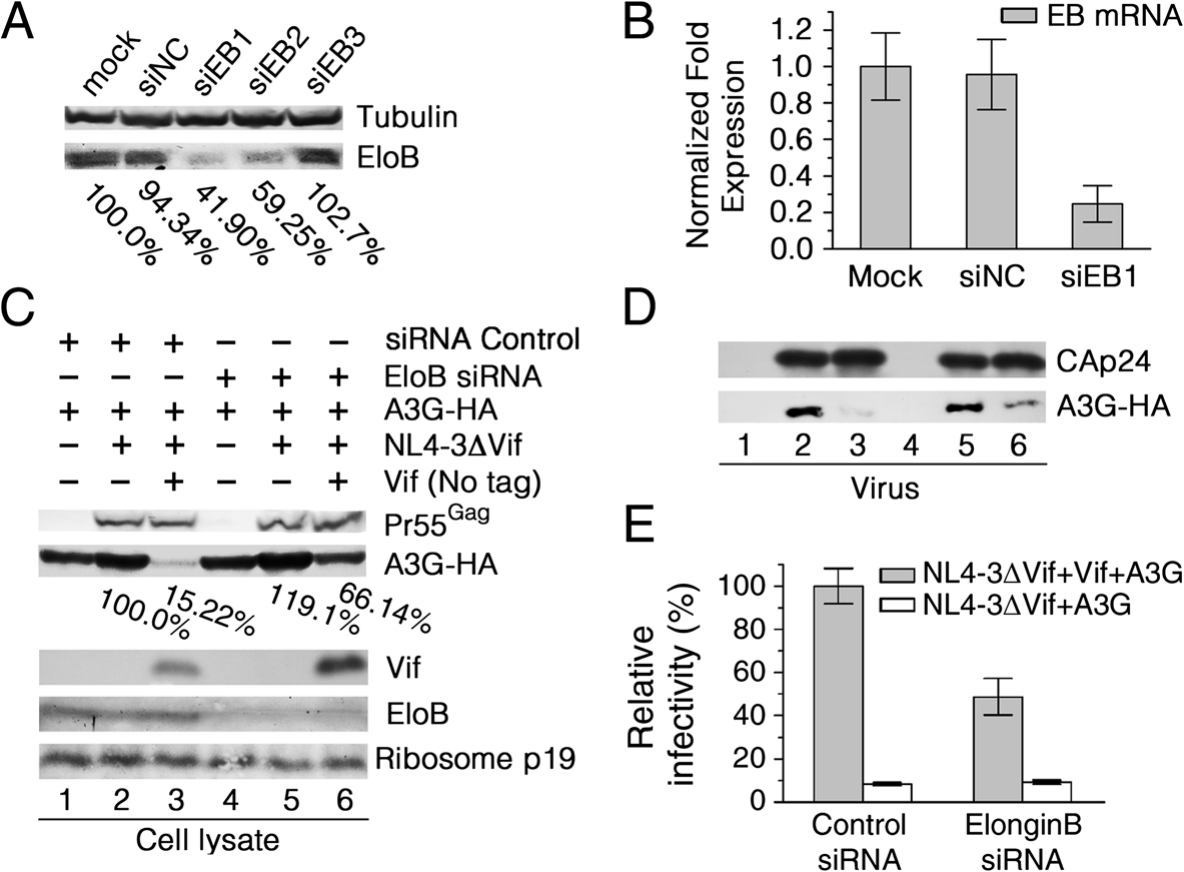
**RNAi-mediated silencing of endogenous EloB expression impairs Vif functions. (A)** 293 T cells were seeded in 6-well plates at 0.8 × 10^6^ cells per well just prior to transfection with siRNAs at a final concentration of 20 nM. The cells were harvested at 72 h post-transfection, and EloB protein expression was analyzed by Western blot. Mock, cells treated with Hiperfect transfection reagent only; siNC, cells transfected with AllStars Negative Control siRNA; siEB1-3, cells transfected with EloB-siRNA1-3. Band intensities were quantified using Bandscan software, normalized to tubulin and expressed as percentages of the mock value. **(B)** 293 T cells transfected with siRNAs were harvested at 72 h post-transfection and analyzed by qRT-PCR for *EloB* mRNA levels normalized to β-actin mRNA. Fold changes in *EloB* gene expression relative to that of the mock control are shown. **(C)** At 24 h after siRNA treatment, 293 T cells (siRNA-treated cells were grown in 25 cm^2^ flasks to about 80% confluency) were co-transfected with 450 ng of VR-A3G-HA, 3.5 μg of NL4-3ΔVif, and 1.2 μg of VR-Vif or VR1012. After 48 h of transfection, cells were examined for A3G expression (lanes 1–3, control siRNA treated cells; lanes 4–6, EloB siRNA treated cells), and corresponding supernatants were used in **(D)** and **(E)**. **(D)** Pelleted virions obtained from **(C)** were examined for A3G–HA virion packaging. **(E)** Relative infectivity of viruses obtained from **(C)** was assessed by the MAGI assay, with the infectivity of viruses produced from cells co-transfected with NL4-3ΔVif and VR-Vif in the presence of control siRNA was set to 100%. Virus input was normalized according to the p24 level. Error bars in **(B)** represent standard deviations from three independent experiments and in **(E)** from triplicate wells.

CBF-β and EloB-EloC are reported to regulate Vif function by promoting folding of different domains of Vif [25,28,34,51,52], both of which affect Cul5 binding to Vif [13,36], while knockdown of CBF-β does not impair EloB-EloC binding to Vif [36]. In order to further study the events in the assembly of the Vif-CBF-β-Cul5 complex, we attempted, but failed, to obtain a complete knockdown of EloC (data not shown), similar to the results obtained by Hwang *et al*. [53]. Therefore, we designed three EloB-specific small interfering RNAs (siRNAs), designated siEB1, 2 and 3, to knock down endogenous EloB expression. Down-regulation of EloB expression in 293 T cells by the three siRNAs was confirmed by Western blot analysis 72 h after transfection (Figure 1A). The EloB protein band intensities in the immunoblot were quantified using Bandscan software (Glyko, Novato, CA) and normalized to tubulin. SiEB1 caused an approximately 60% decrease, whereas siEB2 caused a modest decrease and siEB3 showed nearly no effect, in EloB expression compared with cells transfected with the negative control siRNA (siNC) or those that were mock-transfected (treated with Hiperfect transfection reagent only) (Figure 1A). The silencing efficiency of the most effective EloB siRNA, siEB1, in 293 T cells was further validated by quantitative real-time PCR (qRT-PCR), which showed an approximately 70% decrease in *EloB* mRNA (Figure 1B). Expression levels of EloB mRNA and proteins in these assays were normalized to the expression of β-actin in the corresponding samples. Based on these results, siEB1 was utilized in all subsequent experiments to transiently knock down EloB.

To determine whether EloB knockdown would result in impairment of HIV-1 Vif function, we co-transfected pNL4-3ΔVif and VR-Vif (without a tag) or empty plasmid VR1012 in the presence of siEB1 or siNC. In the siNC-transfected 293 T cells, the over-expressed Vif could efficiently reduce the intracellular level of A3G (Figure 1C, lane 3) when compared to the no Vif control (lane 2). Silencing of endogenous EloB (Figure 1C, lanes 4–6) indeed impaired Vif-mediated depletion of A3G, even in the presence of other HIV-1 proteins (Figure 1C, lane 6). This result confirmed that EloB was required for the Vif-mediated destruction of A3G as reported previously [13,27] (Figure 1C, compare lanes 2, 3 to lanes 5, 6).

Incorporation of A3G into HIV-1 virions is a prerequisite for its antiviral activity [14,54]. Vif circumvents this antiviral activity by depleting intracellular stores of A3G, thereby inducing virion exclusion of A3G. Consistent with other studies [13,33], we found that A3G could be incorporated into HIV-1 virions in the absence of Vif (Figure 1D, lane 2), and when Vif was present, the intravirion packaging of A3G was prevented in siNC-transfected cells (Figure 1D, lane 3). Nevertheless, when using siEB1-transfected cells in which endogenous EloB expression was efficiently suppressed as virus-producing cells, the ability of Vif to block the incorporation of A3G into the budding virus was impaired (Figure 1D, compare lanes 3 and 6). Silencing of endogenous EloB could also impair the ability of Vif to neutralize the antiviral activity of A3G (Figure 1E).

### EloB is critical for the interaction between Vif and CBF-β

**Figure 2:**
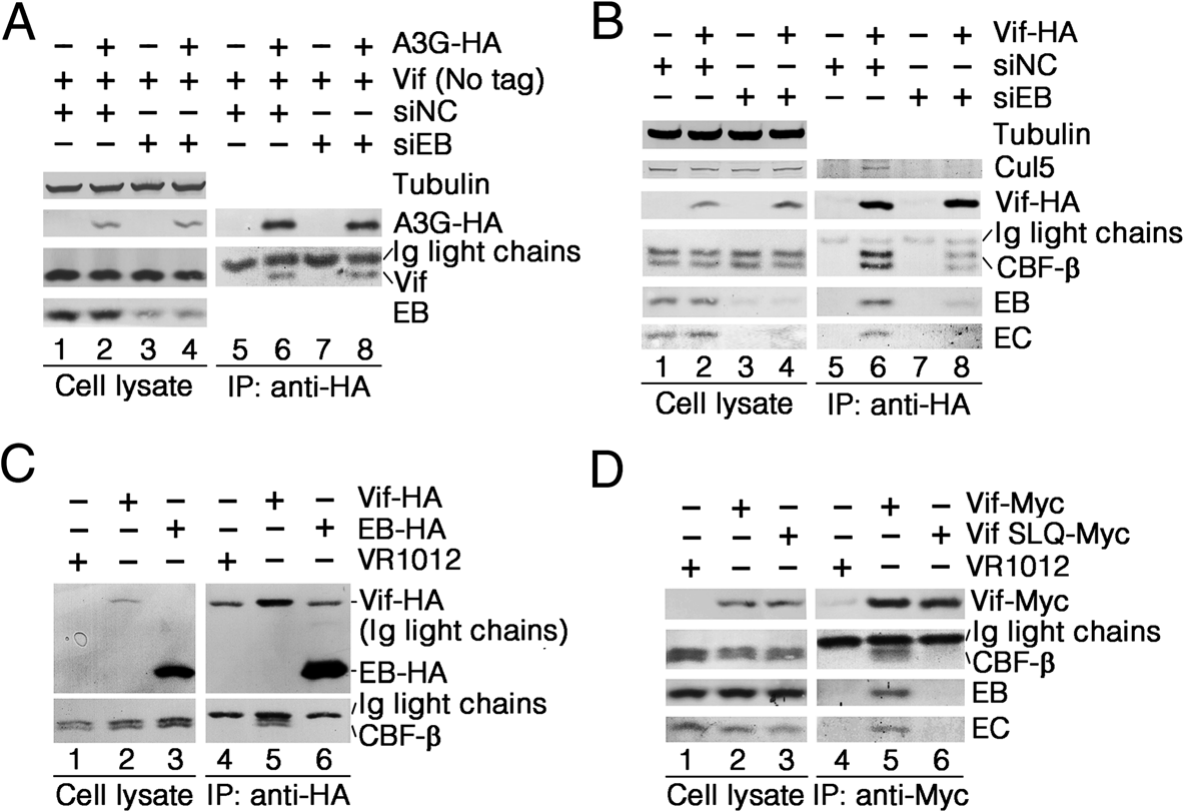
**EloB is critical for the interaction between Vif and CBF-β. (A)** EloB did not affect the interaction between Vif and A3G. 293 T cells (10^6^) were transfected with the indicated siRNAs. At 24 h after siRNA transfection, cells were co-transfected with 2.5 μg of VR-Vif and 1 μg of VR-A3G-HA or VR1012. The cells were treated with MG132 (10 μM) for 12 h before harvesting. Cell lysates were co-immunoprecipitated (Co-IP) with anti-HA antibody, followed by SDS–PAGE and immunoblot analysis. **(B)** EloB silencing impaired both the Vif-CBF-β and the Vif-Cul5 interactions. SiRNA-treated cells (10^6^) were transfected with 2.5 μg of VR-Vif-HA or VR1012. Cell lysates were immunoprecipitated (IP) with anti-HA antibody, followed by SDS–PAGE and immunoblot analysis. **(C)** Endogenous CBF-β co-immunoprecipitated with Vif–HA but not EloB–HA (EB-HA). 293 T cells (10^6^) were transfected with 2.5 μg of VR-Vif-HA or VR-EloB-HA or VR1012. Cell lysates were immunoprecipitated with an anti-HA antibody, followed by SDS–PAGE and immunoblot analysis. **(D)** CBF-β, EloB (EB) and EloC (EC) co-immunoprecipitated with Vif–Myc but not Vif SLQ–Myc. 293 T cells (10^6^) were transfected with 2 μg of VR-Vif-Myc, 2.5 μg VR-Vif SLQ-Myc or VR1012. Cell lysates were immunoprecipitated with an anti-Myc antibody, followed by SDS–PAGE and immunoblot analysis. All results are representative of three independent experiments.

Because it was recently reported that EloB interacts with the PPLP motif of Vif which is necessary for A3G binding [26,55], we considered whether knockdown of endogenous EloB expression would interfere with the interaction between A3G and Vif. To determine whether EloB is required for Vif binding to A3G, a vector expressing untagged Vif or empty vector VR1012 was co-transfected with VR-A3G-HA into 293 T cells for a co-immunoprecipitation assay. The transfected cells were treated with the proteasome inhibitor MG132 (10 μM) for 12 h before harvesting. Vif was efficiently co-immunoprecipitated with A3G–HA by the anti-HA antibody both in siNC-transfected cells and in siEB1-transfected cells (Figure 2A, compare lanes 6 and 8), showing that the reduction of EloB expression had no effect on the Vif-A3G interaction. Therefore, we concluded that EloB may not regulate substrate recognition of Vif.

To explore if silencing of EloB could affect the expression of E3 components, or their interactions with Vif, we transfected a vector expressing HA-tagged Vif into 293 T cells treated with siNC or siEB1, followed by co-immunoprecipitation. Indeed, expression levels of Cul5 and CBF-β were not affected by silencing of endogenous EloB expression (Figure 2B, compare lanes 1, 2 to lanes 3, 4). However, a significant decrease in CBF-β binding to Vif-HA was observed when EloB was silenced compared to the siNC-treated cells (Figure 2B, compare lanes 6 and 8). It is reasonable that the levels of EloC were greatly reduced in siEB1-transfected cells, which is consistent with the results obtained by Hwang *et al*. [53], as EloB is known to stabilize EloC and form an obligate heterodimer with EloC [13,38-42]. It was not surprising to find that the interaction between Cul5 and Vif–HA was significantly disrupted in siEB1-transfected cells, since failures of both CBF-β and EloB-EloC in binding to Vif have been reported to block the Vif-Cul5 interaction [27,33]. The results indicated that EloB may affect the function of Vif by influencing assembly of the Vif-Cul5 E3 complex. However, the remaining question to be resolved in our study is whether these effects are caused by the absence of EloB or EloC (or both).

Zhang *et al*. had reported that CBF-β does not interact with Cul5 or EloB/C [33]. To reconfirm that there was no direct interaction between EloB and CBF-β, vectors expressing EloB or Vif were transfected into 293 T cells. Both proteins were fused to an HA epitope at their C-terminus, and their expression levels were observed in 293 T cells (Figure 2C, lanes 2, 3). Both EloB and Vif could be immunoprecipitated by the anti-HA antibody (Figure 2C, lanes 5, 6), and CBF-β was efficiently co-immunoprecipitated with Vif (Figure 2C, lane 5). Indeed, CBF-β was not co-immunoprecipitated with EloB (Figure 2C, lane 6).

After determining that silencing of EloB expression could impair binding of Vif to CBF-β, we postulated that the interaction of Vif with EloB (and EloC) is important for Vif-CBF-β binding. To verify this hypothesis, we transfected vectors expressing Vif or Vif-SLQ into 293 T cells. Both of these expressed proteins were tagged with a C-terminal Myc epitope. Vif-SLQ contains mutations of critical residues in the Vif SOCS box (SLQ to AAA), which can significantly disrupt the interaction of mutant Vif with EloB-EloC. The results showed that Vif and Vif-SLQ were expressed at comparable levels (Figure 2D, lanes 2 and 3), and both of these proteins could be immunoprecipitated by the anti-Myc antibody (Figure 2D, lanes 5 and 6). CBF-β could be efficiently co-immunoprecipitated with Vif, but it showed almost no interaction with Vif-SLQ (Figure 2D, compare lanes 5 and 6). This result indicated that the Vif-EloB-EloC interaction is important for Vif-CBF-β binding.

### Over-expression of EloB prevents significant degradation of Vif

**Figure 3:**
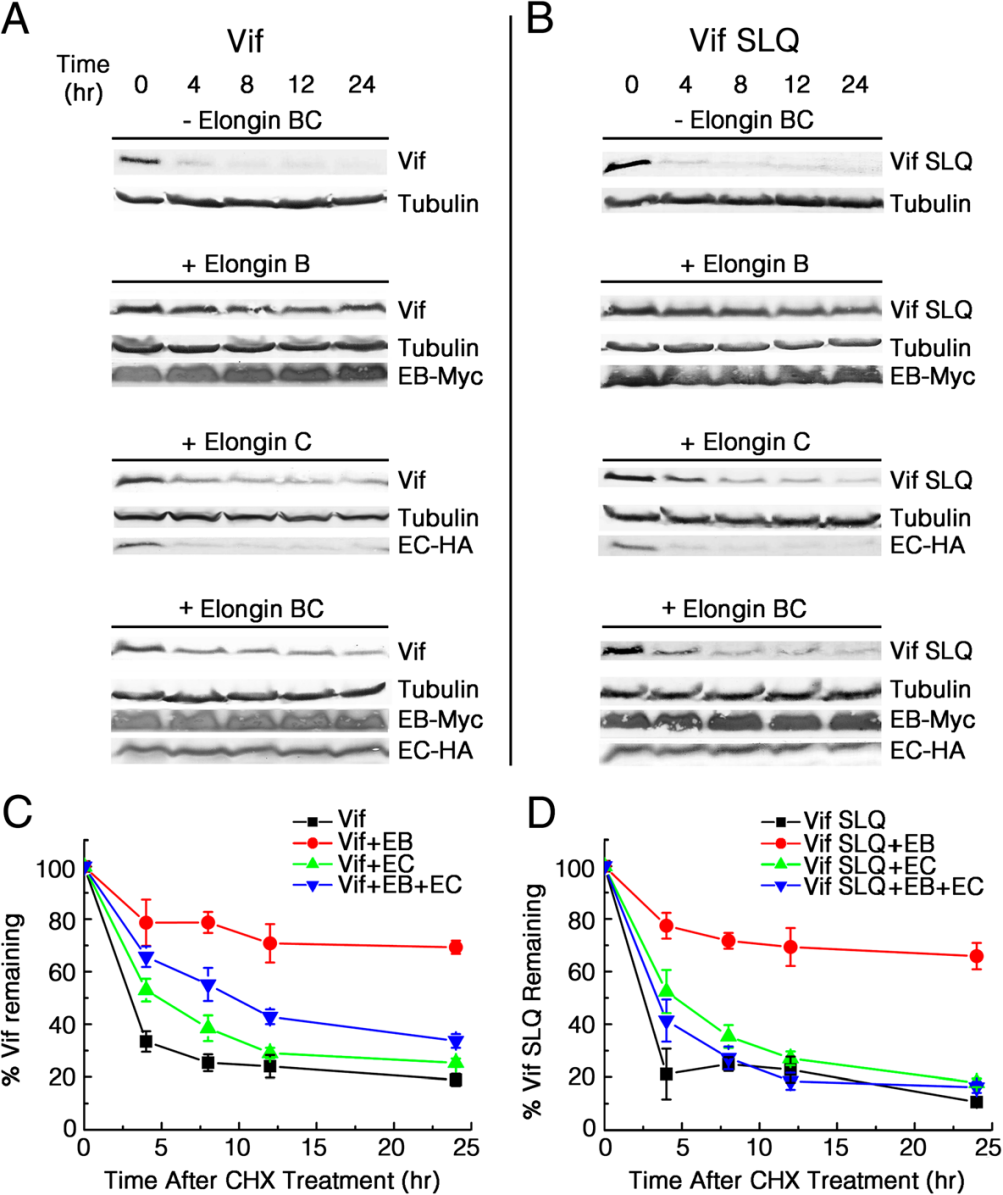
**EloB significantly stabilizes Vif.** 293 T cells (10^6^) were cotransfected with 1 μg of VR-Vif or VR-Vif-SLQ, either without (- Elongin BC) or with co-transfection of 1 μg VR-EloB (+ Elongin B), 1 μg VR-EloC (+ Elongin C), or both 1 μg VR-EloB and 1 μg VR-EloC (+ Elongin BC). Cells were treated with 100 μg/ml CHX 36 h after transfection and harvested at the indicated time points. Western blot analysis of Vif/Vif-SLQ (upper panels) was performed using an anti-Vif antibody. EloB-Myc (EB-Myc) and EloC-HA (EC-HA) were detected with appropriate epitope-tag antibodies (lower panels). **(A)** Positions of Vif are indicated on the right (upper). **(B)** Positions of Vif-SLQ are indicated on the right (upper). **(C)** Individual band intensities of Vif in **(A)** were quantified using Bandscan software, normalized to the quantity of tubulin bands, expressed as percentages of the value at 0 h (set to 100%) and graphically represented. **(D)** Quantification of Vif-SLQ bands in **(B)** was performed as described in **(C)**. All results are representative of three independent experiments. Error bars represent ± SD (n = 3).

HIV-1 Vif itself has been reported to undergo rapid proteasomal degradation by MDM2 and other E3 ligases [56,57]. As VHL is known to protect against proteasomal degradation by binding with EloB-EloC [58], we speculated that the binding of EloB (and EloC) to Vif may affect the stability of Vif . To test this hypothesis and to assess the effect of EloB and EloC binding on Vif stability, we transiently co-transfected the untagged Vif or VifΔSLQ expression vector into 293 T cells in the absence or presence of EloB or EloC expression vector or both and examined the stability of Vif/Vif-SLQ molecules using the cycloheximide (CHX) stability assay (Figure 3). To determine relative Vif expression levels, band intensities detected in this assay were scanned using the Glyko Bandscan software and normalized to tubulin. As previously reported [56], over-expressed wild-type Vif and Vif-SLQ were both rapidly degraded in the absence of EloB-EloC (Figure 3). Co-transfection of both EloB and EloC could increase Vif stability but showed almost no effect on Vif-SLQ degradation (Figure 3). These results demonstrated that EloB-EloC binding could stabilize Vif. Strikingly, co-transfection of EloB and Vif/Vif-SLQ markedly stabilized both wild-type Vif and Vif-SLQ to a greater extent than did co-transfection of EloB-EloC, whereas co-transfection of EloC and Vif/Vif-SLQ only slightly increased the stability of wild-type Vif and Vif-SLQ (Figure 3).

The finding above (Figure 3) was different from another study showing that EloB has almost no effect on VHL stability [58], which suggests that EloB may have more functions in the Vif-Cul5 E3 complex than it does in the VHL-Cul2 E3 complex. Additionally, the results seemed to be contradictory to those in Figure 1C and Figure 2A. That is, EloB could stabilize the Vif protein in our experiments, but when EloB expression was silenced by siRNA, the levels of Vif protein were not affected in Figure 2A, while they were increased in Figure 1C. We presumed that EloB may have prevented the degradation of Vif through the same sites by which it interacted with EloC, and thus only over-expression of EloB (free EloB) could result in Vif stabilization. In other words, when EloB was co-expressed with EloC, it may have preferentially formed an obligate heterodimer with EloC and lost the ability to significantly stabilize Vif. Therefore, the endogenous EloB in the cell could not stabilize Vif in the presence of endogenous EloC and would not cause Vif downregulation when it was knocked down. Moreover, as it is generally believed that A3G and Vif are both degraded by the EloB-EloC-Cul5 E3 ligase [15,59-61], it may be reasonable that the decrease of EloB (and EloC) protein level which would hinder the formation of the E3 complex could allow both Vif and A3G to escape from degradation (Figure 1C). Therefore, it is not contradictory to find that EloB over-expression stabilizes Vif and that down-regulation of endogenous EloB also stabilizes Vif. The discrepancy between Figure 1C and Figure 2A may be due to the addition of MG132 at 12 h before cell harvesting in Figure 2A.

We also observed that the Vif-SLQ mutant did not have a longer half-life than the wild-type Vif protein, which seemed to be paradoxical if Vif was degraded by the EloB-EloC-Cul5 E3 ligase [15,59-61]. However, it was reported that coexpression of A3G promotes polyubiquitination and proteasomal degradation of Vif [61]. That is, in the absence of A3G, the EloB-EloC-Cul5 E3 ligase would have little effect on the half-life of Vif [61]. Furthermore, Vif was also reported to be degraded by the MDM2 E3 ligase and other unknown mechanisms [57]. Thus, it was reasonable that the Vif-SLQ mutant showed a similar half-life to that of the wild-type Vif protein. Moreover, without assistance of EloB-EloC, the Vif-SLQ mutant may fold differently than the wild-type Vif, which may also affect its stability.

### Dissecting the interaction network of Vif-CBF-β-EloB-EloC complex subunits by co-expression in *E*. *coli*

**Figure 4:**
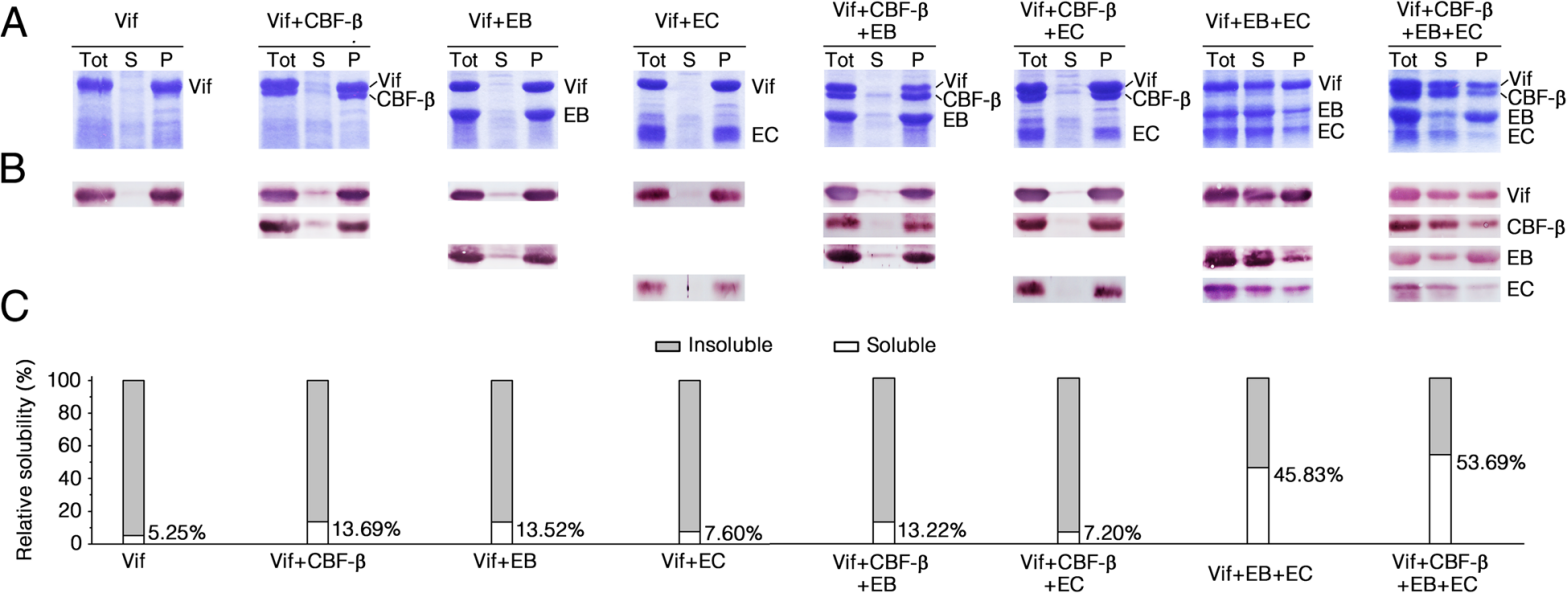
**Soluble Vif and CBF-β proteins can be efficiently produced only in the presence of EloB/C.***E*. *coli* BL21 (DE3) cells containing appropriate pST39 constructs were induced, harvested, homogenized by sonication and then clarified by centrifugation. For solubility analysis, the supernatant and the pellet were subjected to SDS–PAGE. All proteins were untagged. **(A)** Solubility of Vif, expressed alone or co-expressed with CBF-β, EloB, EloC, CBF-β + EloB, CBF-β + EloC, EloB + EloC or CBF-β + EloB + EloC, was determined by visualization with Coomassie staining. Tot, total cell lysate; S, supernatant; P, pellet. **(B)** Fractions in **(A)** were visualized by immunoblotting using appropriate antibodies. **(C)** Quantification of band intensities of Vif in **(B)**. All experiments were repeated three times.

Co-expression of subunits of multiprotein complexes in *E*. *coli* has been used for interaction analysis [51,62]. Successful over-expression of soluble proteins is thought to be achieved if the subunit proteins are co-expressed with their cognate partners [63-65]. In other words, subunits of a complex may fold incorrectly when expressed individually, but they may mutually enhance the folding of co-expressed proteins, thereby preventing aggregation of nascent unfolded proteins and leading to increase solubility. To co-express components of the Vif-CBF-β-EloB-EloC complex, we used the pST39 polycistronic expression system, which allowed simultaneous over-expression of the subunits [63]. To rule out potential effects of epitope tags on Vif solubility, all subunits were expressed untagged. We found that the full-length Vif protein (residues 1–192) was predominantly insoluble (>90%) as determined by both Coomassie staining and immunoblotting with a Vif-specific antibody (Figure 4). Co-expression with full-length CBF-β (isoform 1, residues 1-182), EloB or EloC could not substantially improve the solubility of Vif (Figure 4). This result indicated that CBF-β, EloB and EloC individually may not be able to interact with Vif in the absence of other E3 components. Co-expression of Vif with both CBF-β and EloB did not improve its solubility when compared with that when co-expressed with EloB only; likewise, co-expression of Vif with both CBF-β and EloC also did not increase its solubility compared with that when co-expressed with EloC alone (Figure 4). These results indicated that CBF-β could not co-fold well with Vif even in the presence of EloB or EloC. However, when Vif was co-expressed with both EloB and EloC, its solubility was significantly increased and was modestly improved by co-expression with CBF-β (Figure 4). The solubility of CBF-β also could only be improved by co-expression with Vif-EloB-EloC. These results indicated that the co-folding of CBF-β and Vif may be dependent on the interaction of Vif with both EloB and EloC *in vitro*, which was basically consistent with results of our co-immunoprecipitation analysis (Figure 2B, D).

This experiment had some similarities to an analysis performed by Zhou *et al*. [51], in which there seemed to be a discrepancy regarding the effect of CBF-β on Vif solubility. The authors used a truncated His-tagged CBF-β (residues 1–140), instead of an untagged full-length CBF-β as in the current study, to evaluate the effect of CBF-β on Vif solubility. Since the notion is generally accepted that the solubility of an over-expressed recombinant protein in *E*. *coli* can be greatly affected by slightly changing the amino acid sequence at its N- or C-terminus [66-69], it was reasonable that our results differed from each other. Zhou *et al*. also reported that the purified Vif-CBFβ140 complexes were aggregated and unstable [51]. In their study, the Vif-CBFβ140 complexes precipitated quickly at low temperatures, with the Vif protein precipitating even faster than the CBFβ140 protein, while the Vif-CBFβ140-EloB-EloC complexes were much more stable [51]. Their results indicated that the Vif-CBFβ140 complexes may be misfolded in the absence of EloB-EloC in *E*. *coli*, and CBF-β could co-fold well with Vif when they were coexpressed with EloB-EloC, supporting our data.

### Over-expression of EloB mutant with a 34-residue deletion in the COOH-terminal tail blocks Vif-induced A3G degradation

**Figure 5:**
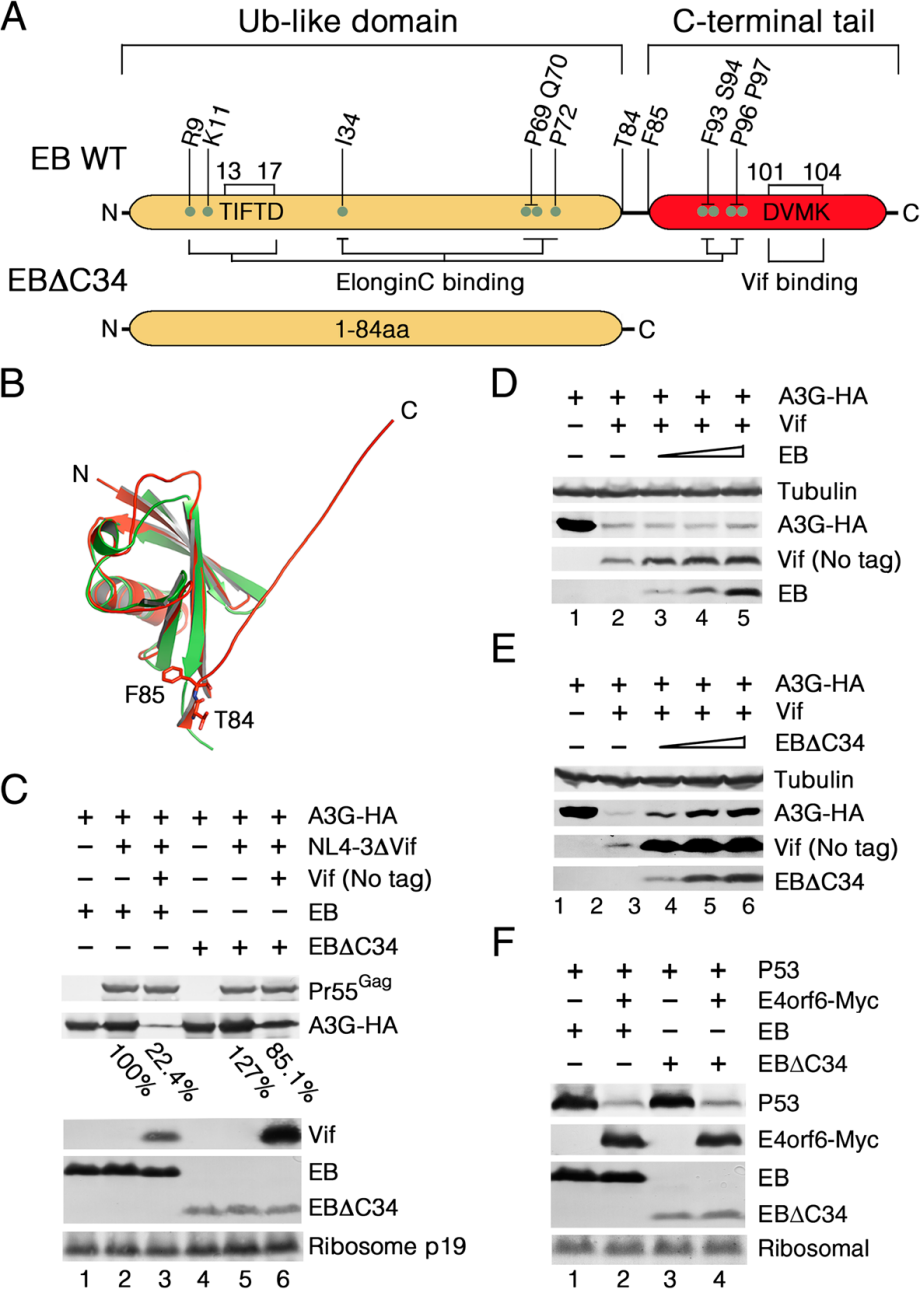
**The 34-amino acid C-terminal tail of EloB is required for regulation of Vif function. (A)** Schematic representations of functional domains of EloB based on existing evidence and of the EloB C-terminal deletion mutant used in this study. **(B)** Structural alignment of EloB (red) and ubiquitin (green). The tertiary structure of the EloB UbL domain bears striking similarity to that of ubiquitin, whereas the tertiary structure of the EloB C-terminal tail was not found in that of ubiquitin. **(C)** 293 T cells grown in 25 cm^2^ flasks to about 80% confluency were co-transfected with 450 ng of VR-A3G-HA, 3.5 μg of NL4-3ΔVif, and 1.2 μg of VR-Vif or VR1012 in the presence of 750 ng of wild-type EB (lanes 1–3) or 1.5 μg of EBΔC34 (lanes 4–6). Cells were harvested 48 h after transfection and analyzed by Western blot. A3G expression was calculated relative to that detected in the absence of both Vif and EBΔC34 (set to 100%). **(D)** 293 T cells (10^6^) were co-transfected with 300 ng of VR-A3G-HA, 600 ng of VR-Vif or VR1012 and increasing amounts of vectors expressing EB or VR1012 (150, 250 and 350 ng). Cells were harvested 48 h after transfection and analyzed by Western blot. **(E)** 293 T cells (10^6^) were co-transfected with 300 ng VR-A3G-HA, 600 ng VR-Vif or VR1012 and increasing amounts of vectors expressing EBΔC34 or VR1012 (300, 500 and 700 ng). Cells were harvested 48 h after transfection and analyzed by Western blot. **(F)** 293 T cells (10^6^) were co-transfected with 7.5 ng VR-P53-HA and 1 μg VR-E4orf6-Myc or VR1012 in the presence of 750 ng of wild-type EB (lanes 1–2) or 1.5 μg of EBΔC34 (lanes 3–4). The cells were harvested 48 h post-transfection and analyzed by Western blotting. All experiments were repeated three times.

Several lines of evidence have indicated that the Vif-binding motif is located in the C-terminal tail of the EloB protein, whereas residues involved in the contact with EloC are dispersed throughout the EloB primary sequence in both the N-terminal UbL domain and the C-terminal region (indicated in Figure 5A) [26,48,70,71]. The conserved EloB N-terminal was named the UbL domain because it bears marked sequence similarity to ubiquitin [48], and its tertiary structure (shown in red, PDB code: 3DCG) is strikingly similar to that of ubiquitin (shown in green, PDB code: 1UBQ) when aligned using software PyMol (http://pymol.org/, Figure 5B) [28,72]. The conserved UbL domain is reported to be necessary and sufficient for the EloB-EloC interaction [47,48], the function of which has been well-studied, but the C-terminal region of EloB is also highly conserved. The recently discovered interaction between the C-terminus of EloB and Vif implied that there may be as yet undiscovered functions of EloB within the Vif-Cul5 E3 complex.

To explore the possible functions of the EloB C-terminal domain, we constructed a mutant with a deletion of 34 amino acids in the C-terminal tail, EBΔC34 (Figure 5A), which was untagged to rule out potential effects of an epitope tag on EB function. To determine whether the EloB C-terminal deletion would affect Vif function, 293 T cells were co-transfected with pNL4-3ΔVif and VR-Vif (without a tag) or empty plasmid VR1012 in the presence of VR-EloBΔC34 or VR-EloB. The untagged EBΔC34 and wild-type EB were detected by immunoblotting with an anti-EloB polyclonal antibody, which was raised using a synthetic peptide derived from the internal sequence (residues 20–48) of human EloB (Abcam, NP_009039.1). The Vif-mediated depletion of A3G was impaired by over-expression of EBΔC34 (Figure 5C) and this impairment was dose-dependent (Figure 5E), whereas wild-type EB had no effect of impairment (Figure 5C, D). These results indicated that the C-terminal tail of EloB was important for EloB to improve Vif function.

**Figure 6:**
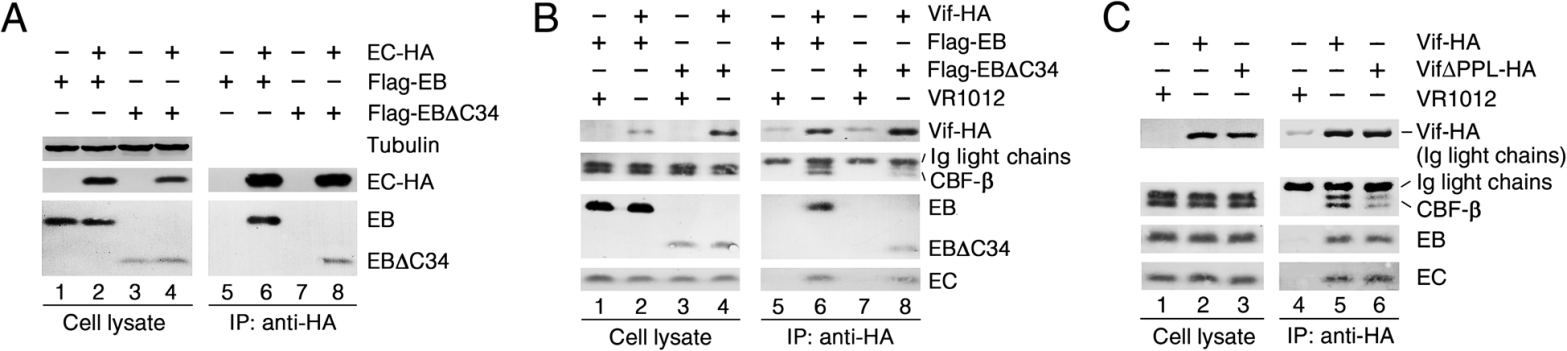
**EloB promotes CBF-β binding to Vif mainly by its C-terminus. (A)** Both Flag-tagged EB and EBΔC34 co-immunoprecipitated with EC-HA. 293 T cells (10^6^) were co-transfected with 1 μg of VR-EC-HA and 600 ng of VR-Flag-EB or 3 μg of VR-Flag-EBΔC34. Cell lysates were immunoprecipitated with anti-HA antibody, followed by SDS–PAGE and analyzed by Western blotting with anti-HA, anti-Flag and anti-tubulin antibodies. **(B)** Over-expression of Flag-tagged EBΔC34 impaired the interaction between Vif and CBF-β. 293 T cells (10^6^) were transfected with 2 μg of VR-Vif-HA and 400 ng of VR-Flag-EB-HA or 2 μg of VR-Flag-EBΔC34. Cell lysates were immunoprecipitated with an anti-HA antibody, followed by SDS–PAGE and immunoblot analysis. **(C)** The PPL → AAA mutation of Vif resulted in impaired CBF-β binding to Vif. 293 T cells (10^6^) were transfected with 800 ng of VR-Vif-HA or 3 μg of VR-VifΔPPL-HA or VR1012. Cell lysates were immunoprecipitated with an anti-HA antibody, followed by Western blotting with anti-HA, anti-CBF-β, anti-EloB and anti-EloC antibodies. The result is representative of three independent experiments.

Interestingly, the co-expression of EBΔC34 strikingly increased the intracellular protein level of Vif, even at a low dose (Figure 5C, E), indicating a possible stabilization of Vif by EBΔC34. The over-expression of EBΔC34 did not affect the endogenous expression of EloC (Figure 6B), ruling out the possibility that EloB regulates Vif function completely by affecting the stability of EloC and settling the question posed above.

To determine whether over-expression of the dominant-negative mutant EBΔC34 is capable of blocking poly-ubiquitination of all substrates, we employed the adenovirus protein E4orf6 because it contains a Vif-like BC-box by which it assembles with Cul5-EloB-EloC E3 complex, similar to Vif, to regulate p53 [73]. 293 T cells were co-transfected with VR-P53-HA and E4orf6-pCMV6.9 or empty plasmid VR1012 in the presence of VR-EloBΔC34 or VR-EloB. The harvested cells were analyzed by Western blotting with anti-P53, anti-myc, anti-EloB and anti-ribosomal antibodies. According to the results, the over-expression of EBΔC34 had almost no effect on the E4orf6-mediated depletion of P53 (Figure 5F), even when the amount of expression plasmid was increased (data not shown), implying that the C-terminal tail of EloB may be specifically required for improving Vif function.

### The interaction between the Vif PPLP motif and the conserved 34-amino acid COOH-terminal tail of EloB plays a role in promoting recruitment of CBF-β to the Vif-Cul5 E3 complex

Since the C-terminal tail of EloB also contains residues that are involved in EloC binding [71] (Figure 5A), we further examined whether EloBΔC34 could still interact with EloC. 293 T cells were transfected with VR-EloC-HA and VR-Flag-EloB or VR-Flag-EloBΔC34 to examine the interaction between HA-tagged EloC and N-terminally flag-tagged EloB or EloBΔC34. The cell lysates were immunoprecipitated with an anti-HA antibody. We found that both EloB and EloBΔC34 could be efficiently co-immunoprecipitated with EloC-HA (Figure 6A), indicating a similar ability to bind EloC. To explore whether EBΔC34 may influence Vif function by affecting assembly of the Vif-Cul5 E3 complex as observed with EloB silencing, we examined the interaction of Vif-HA with endogenous CBF-β and EloC in the presence of VR-flag-EBΔC34 or VR-flag-EB by co-immunoprecipitation analysis. The results showed that the CBF-β binding to Vif was impaired by over-expression of the N-terminally Flag-tagged EloBΔC34, whereas the interaction of EloC with Vif was not apparently affected (Figure 6B), indicating that the 34-amino acid C-terminal tail of EloB may play a role in promoting the recruitment of CBF-β to Vif. EloB/EloBΔC34 itself could also be co-immunoprecipitated with Vif-HA (Figure 6B).

Since the C-terminus of EloB interacts with the PPLP motif of Vif [26], we wondered whether the PPL → AAA mutation of Vif could also result in impaired CBF-β binding to Vif. To test this hypothesis, we transfected vectors expressing Vif or VifΔPPL into 293 T cells. Both of the expressed proteins were tagged with a C-terminal HA epitope. The results showed that both Vif and VifΔPPL could be immunoprecipitated by the anti-HA antibody (Figure 6C), but VifΔPPL appeared to be less able to bind CBF-β (Figure 6C). This result indicated that the C-terminus of EloB may affect Vif-CBF-β binding by interacting with the PPLP motif of Vif and inducing its structural change.

### EloB stabilizes Vif mainly through residues 9-14 within its ubiquitin- homology domain

**Figure 7:**
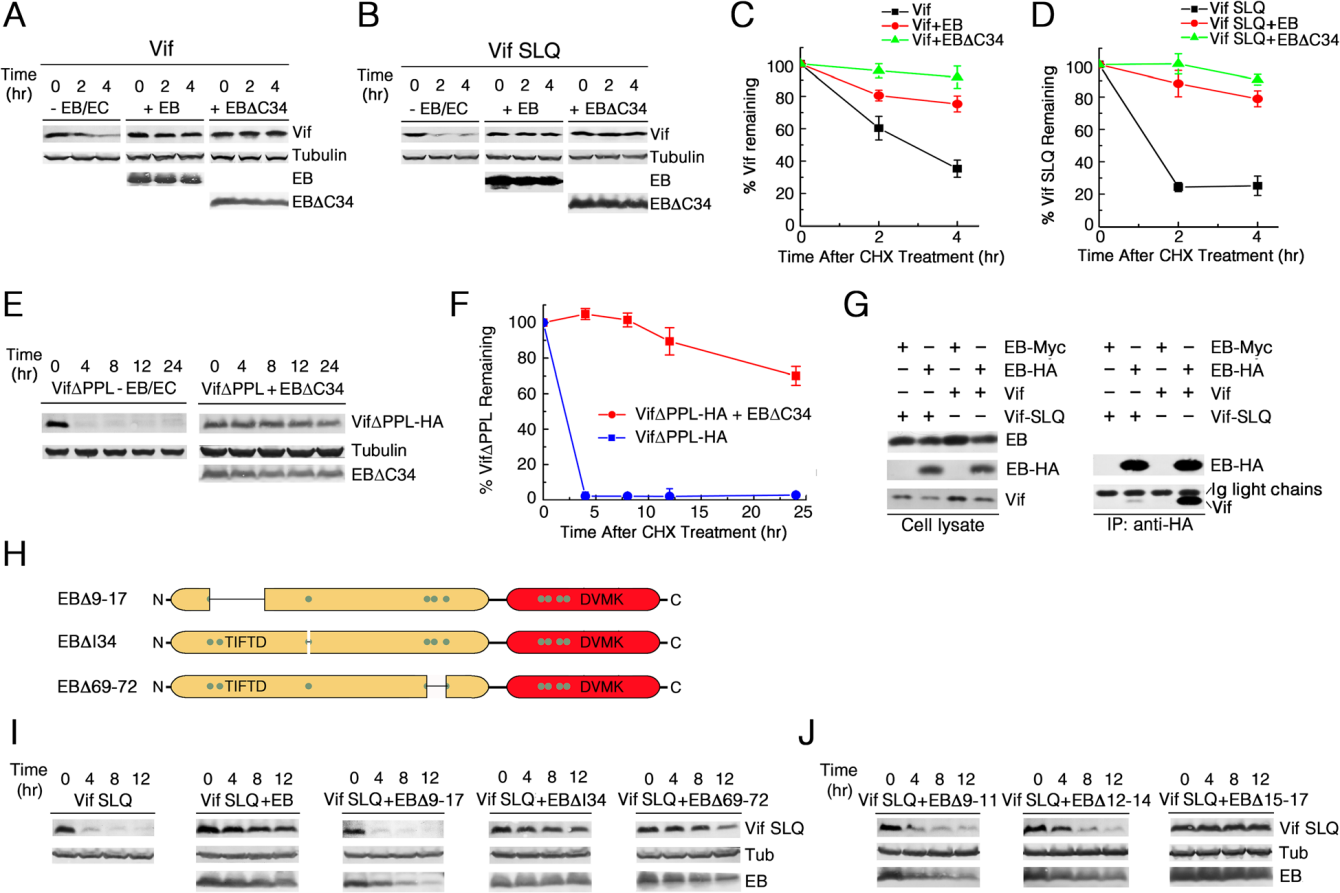
**EloB stabilizes Vif via the same region by which it interacts with EloC. (A, B)** Vectors expressing untagged Vif (1 μg) or Vif-SLQ (1 μg) were transiently transfected into 293 T cells (10^6^), either without (-EB/EC) or with co-transfection of 1 μg of EloB (+EB) or 2 μg of EloBΔC34 (+EBΔC34). Cells were treated with CHX and harvested as described in Figure 3. **(C, D)** Quantification of Vif/Vif-SLQ bands in **(A, B)**. **(E)** Vectors expressing VifΔPPL-HA (1 μg) were transiently transfected into 293 T cells (10^6^), either without (-EB/EC) or with co-transfection of 2 μg of EloBΔC34 (+EBΔC34). Cells were treated with CHX and harvested. **(F)** Quantification of VifΔPPL-HA bands in **(F)**. All results in **(C, D, F)** are representative of three independent experiments. Error bars represent ± SD (n = 3). **(G)** 293 T cells (10^6^) were co-transfected with 1 μg of VR-EB-HA or VR-EB-Myc and 2.5 μg VR-Vif-SLQ or 1 μg VR-Vif. Cell lysates were co-immunoprecipitated with an anti-HA antibody, followed by Western blotting with anti-HA, anti-EloB and anti-Vif antibodies. Results are representative of six independent experiments. **(H)** Schematic representation of the deletion mutagenesis of the EloC binding domain of EloB. **(I)** 293 T cells (10^6^) were transfected with 1 μg of VR-Vif-SLQ, either without or with co-transfection of 1 μg VR-EloB-HA (+EB), VR-EloBΔ9-17-HA (+EBΔ9-17), VR-EloBΔI34-HA (+EBΔI34) or VR-EloBΔ69-72-HA (+EBΔ69-72). Cells were treated with CHX and harvested. Vif-SLQ were detected with anti-Vif antibody, and EloB was detected with an anti-HA antibody. Results are representative of three independent experiments. **(J)** 293 T cells (10^6^) were transfected with 1 μg of VR-Vif SLQ with co-transfection of 1 μg VR-EloBΔ9-11-HA (+EBΔ9-11), VR-EloBΔ12-14-HA (+EBΔ12-14) or VR-EloBΔ15-17-HA (+EBΔ15-17). Cells were treated with CHX and harvested. Vif-SLQ and EloB was detected as described in **(I)**. Results are representative of three independent experiments.

We had found that over-expression of EloB could stabilize both wild-type Vif and Vif-SLQ. Since the over-expression of EloBΔC34 could increase the intracellular protein level of Vif (Figure 5), we postulated that the UbL domain would be sufficient for EloB to stabilize Vif. Therefore, we over-expressed untagged Vif and Vif-SLQ by transient transfection of their expression vectors into 293 T cells in the absence or presence of EloB or EloBΔC34 plasmids. The stabilities of Vif and Vif-SLQ were assessed by the CHX stability assay. As expected, EloBΔC34 over-expression could stabilize both wild-type Vif and Vif-SLQ, even to a greater extent than with EloB (Figure 7).

Since EloBΔC34, which had lost the PPLP Vif binding motif, could still stabilize Vif [26], we postulated that the stabilization of Vif by EloB might be independent of the PPLP motif. To verify this hypothesis, we transiently transfected vectors expressing VifΔPPL-HA in the absence or presence of EloBΔC34 into 293 T cells and examined the stability of VifΔPPL-HA proteins. The results showed that VifΔPPL could also be significantly stabilized by EloBΔC34 (Figure 7E, F).

As it is generally believed that stabilization should be accompanied by an interaction, we examined the interactions between over-expressed EloB and Vif-SLQ mutants. Indeed, an interaction was found between EloB and the Vif-SLQ mutant, which was much weaker than that between EloB and wild-type Vif (Figure 7G). The Vif-PPL mutant was also able to interact with EloB-EloC (Figure 6C). Therefore, EloB may stabilize Vif-SLQ and Vif-PPL mutants also by binding to them.

In order to explore whether EloBΔC34 could stabilize all SOCS proteins that form complexes with Cul5, we examined the stability of the E4orf6 protein in the absence or presence of EloBΔC34. Unfortunately, the half-life of E4orf6 is very long, and it was found by itself to be very stable without over-expression of EloBΔC34 (data not shown). However, we observed that the protein level of E4orf6 could be slightly down-regulated by over-expression of EloBΔC34 (data not shown).

We have presumed that EloB prevented degradation of Vif through the same sites by which it interacted with EloC. To verify this hypothesis, we constructed three mutants, VR-EBΔ9-17-HA, VR-EBΔI34-HA and VR-EBΔ69-72-HA, each of which contained a deletion in a region in the EloB UbL domain involved in the contact with EloC (Figure 7H), in order to examine their effects on Vif stability. We found that the EBΔ9-17 mutant almost lost the ability to stabilize Vif whereas the EBΔI34 and EBΔ69-72 mutants could still significantly prevents degradation of Vif (Figure 7I), indicating that EloB stabilized Vif through residues 9-17. To further explore the exact site responsible for Vif stabilization, we constructed three mutants, VR-EBΔ9-11-HA, VR-EBΔ12-14-HA and VR-EBΔ15-17-HA, and found that the EBΔ9-11 and EBΔ12-14 mutations had impaired the ability of the molecule to stabilize Vif; meanwhile, the EBΔ15-17 mutant could still significantly stabilize Vif (Figure 7J), implying that EloB stabilizes Vif mainly through residues 9-14 which lies within its UbL domain.

## Discussion

### EloB (and EloC) plays a role in promoting CBF-β binding to Vif

A recently discovered component of the Vif-Cul5 E3 complex, CBF-β, was reported to regulate Vif-Cul5 E3 ligase assembly by promoting folding of the N-terminal region of Vif [33-36], contributing to the Cul5 binding to Vif [13,33,36], which raised further concerns about the functions of components of the Vif-CBF-β-Cul5 complex. In an attempt to investigate functions of the components of the Vif-CBF-β-EloB-EloC-Cul5 E3 ligase, we failed to knock down EloC effectively. However, with the successful knockdown of endogenous EloB expression by using siRNA, it was not surprising to find that indeed Vif function was impaired (Figure 1). To explore the mechanism used by EloB to affect Vif function, we examined the interactions of HIV-1 Vif with its substrate A3G and its cognate partners, endogenous Cul5, EloB/EloC and CBF-β, under the condition of EloB silencing. We found that EloB silencing could reduce Vif binding to CBF-β and Cul5, whereas binding to A3G did not seem to be affected (Figure 2). The decrease in Cul5 binding may have been caused by the decline of Vif-EloC binding and Vif-CBF-β binding, both of which had been reported to negatively affect Cul5 binding to Vif [13,33]. Therefore, we considered that EloB might play a role in influencing the recruitment of CBF-β to Vif. However, since EloC expression was also reduced by EloB silencing (Figure 2), we wondered if EloB could regulate the Vif-CBF-β binding by directly interacting with Vif (or CBF-β) or simply by down-regulating the Vif-EloC interaction, or both.

### Possible mechanisms used by EloB (and EloC) to facilitate CBF-β recruitment

By examining the interaction of CBF-β with EloB, we confirmed that there was no direct contact between them, consistent with the experimental results reported by Zhang *et al*. [33]. By using a VifΔSLQ mutant, we found that the Vif-EloC interaction was crucial for CBF-β binding to Vif. It has been reported that Vif interacts with CBF-β through its N-terminus, whereas the contact sites between EloB-EloC and CBF-β are located in its C-terminus [13,33]. Therefore, EloB and EloC may affect the recruitment of CBF-β to Vif by inducing a structural change in Vif. Based on our results, EloB (and EloC) may use two potential mechanisms to induce a structural change of Vif. The first one is that EloB induces EloC folding to facilitate EloC binding to Vif, which would then induce a structural change in Vif. Alternatively, EloB may directly induce a structural change of Vif via its C-terminus, which interacts with the PPLP motif of Vif [26]. However, as reported previously [33], the interaction between Vif and EloB is dependent on the binding of EloB-EloC to the BC-box of Vif [33]. To evaluate which of these explanations would be more plausible, we constructed a dominant-negative EloB mutant with a deletion of the C-terminal 34 amino acids, which still retains the ability to bind EloC but lacks the Vif binding motif (Figure 5) [26]. We showed that over-expression of the EloBΔC34 mutant (but not the EloBΔC11 mutant, data not shown) could impair Vif function when E4orf6-mediated depletion of P53 seemed not to be affected (Figure 5). The over-expressed EloBΔC34 mutant also reduced the interaction of Vif with CBF-β (Figure 6). The interaction of EloC with Vif was not apparently affected in the presence of EloBΔC34 in our study, implying that the Vif-binding site of EloB may be important for Vif function and for Vif to recruit CBF-β. By using a VifΔPPL mutant, we again found the interaction between Vif and EloB to play a potential role in promoting the Vif-CBF-β interaction, thus lending greater credence to the latter explanation described above. Since the interaction between the Vif PPLP motif and the EloB C-terminus is reported to be required for Vif binding to Cul5 [26], we presumed here that EloB facilitates Vif and Cul5 binding by promoting CBF-β recruitment. However, because the C-terminal 34 amino acids of EloB contains EloC binding sites (Figure 5A) [71], we cannot thoroughly rule out the possibility that EloBΔC34 fails to induce the correct conformation of EloC, thereby influencing EloC-Vif binding and affecting the function of Vif.

Based on our results of the co-immunoprecipitation assays and the analysis of the interaction network of the Vif-CBF-β-EloB-EloC complex in *E*. *coli*, we presumed that EloC alone may not be able to efficiently interact with Vif in the absence of EloB, as mentioned by other researchers previously [74]. In addition, both of the contacts between the Vif BC-box and EloB-EloC and between the PPLP motif of Vif and the C-terminus of EloB can induce a structural change in Vif, as well as facilitate Vif hijacking of cellular CBF-β [26].

### EloB stabilizes Vif utilizing the same region by which it interacts with EloC

Although EloB has previously shown virtually no effect on VHL stability [58], in this study EloB demonstrated an ability to significantly stabilize Vif, and its UbL domain was sufficient to confer this ability (Figure 3, Figure 7). However, we noticed that only over-expression of EloB (free EloB) could remarkably stabilize Vif, while neither the co-expression of EloB and EloC (1:1) nor the native EloB in the cell could result in obvious Vif stabilization (Figures 3, 5, 7). We presumed that this observation may be a result of EloB preventing the degradation of Vif through the same sites by which it interacts with EloC. Thus, when EloB is expressed at comparable levels to EloC, it will preferentially form a stable obligate heterodimer with EloC and lose the capacity to markedly stabilize Vif. This hypothesis was verified by experiments shown in Figure 7. Since a high intracellular protein level of HIV-1 Vif has been shown to inhibit viral infectivity [56], the remarkable stabilization of Vif may also impair viral infectivity. However, the mechanism by which free EloB prevents Vif degradation was not determined in our study.

It is worth noting that stabilization of Vif by EloB had no obvious effects on Vif activity (Figure 5C). When the Vif protein level was greatly increased by EloBΔC34 over-expression, its activity was impaired (Figure 5C, E), suggesting that EloBΔC34 caused a structural aberration or some other distortion that affected the function of Vif. In other words, the amount of the dominant-negative mutant EloBΔC34, which was far in excess of the endogenous EloC, competitively bound to EloC and resulted in the failure to induce a functionally active structure of Vif. This impairment of Vif could be relieved by over-expression of EloC in a dose-dependent manner (data not shown). These results also indicated that EloB uses different pathways to stabilize Vif and to induce structural changes of Vif.

We suspected the possible biological reason in the finding that free EloB can stabilize Vif, especially if there is no consequence on its activity. Most small chaperones could bind transiently to unstructured nascent proteins and thus prevent their premature folding and aggregation [64,75], assisting the proteolytic system to determine the fate of proteins [76]. Thus, although the mechanism that free EloB stabilized Vif was not clear, we supposed that, as a molecular chaperone, free EloB might help to stabilize an intermediate folding state of Vif which has no activity, or it might play a role in the regulation of Vif oligomerization and stabilize a Vif multimer which has no activity, in both of these cases over-expressed EloB could ultimately stabilize the Vif protein level.

We found a weak interaction between the Vif-SLQ mutant and EloB (Figure 7G) which might exist between EloB and the N-terminal region of Vif-SLQ (data was not shown) and result in Vif SLQ stabilization. There seemed to be some some discrepancy regarding the effect of the EloB binding on Vif stabilization. The ELoB interaction with Vif SLQ is much weaker than with wt Vif (as shown in Figure 7G), why is this mutant stabilized as well or even better than wt Vif? Indeed, wt Vif was seemed to have a stronger capability to bind EloB than Vif-SLQ did, but we believe that might be because wt Vif could stably bind EloC which could interact with EloB and induce EloB co-immunoprecipitation with Vif [13]. We could not obtain a complete knockdown of EloC (data not shown), so we were not able to exclude the interference of EloC on estimating the direct interaction between EloB and the N-terminal region of Vif. Thus the direct interaction between EloB and Vif might be comparable with that between EloB and Vif-SLQ, causing the stabilization event of wt Vif as well as or even weaker than that of Vif-SLQ.

## Conclusion

In summary, our results demonstrate that both of the interactions between HIV-1 Vif and human EloB-EloC (Vif SLQ motif with EloC and Vif PPLP motif with EloB) are highly important for the recruitment of CBF-β to Vif. Based on our results, we conclude that EloB can directly affect the assembly of the Vif-CBF-β-Cul5 E3 complex as EloC does by interacting with the PPLP motif of Vif. We identified that the EloB C-terminus, which is not necessary for EloC binding and for E4orf6-mediated depletion of P53, may play a role in improving Vif function. Therefore, the EloB C-terminal tail may be investigated as a promising drug target. Moreover, we found that over-expressed EloB could remarkably stabilize Vif mainly though the same region (residues 9-14) it used to bind EloC. And this important point further expands the diversity of functions of UbL proteins. Thus, our observations extend the current understanding of the function of the Vif-CBF-β-Cul5 E3 ligase.

## Methods

### Plasmids

The polycistronic expression system pST39/pET3aTr (a generous gift from Song Tan, Pennsylvania State University, University Park, PA) was used to produce the plasmids pST39-Vif, pST39-EloB, pST39-EloC, pST39- CBF-β, pST39-Vif-CBF-β, pST39-Vif-EloB, pST39-Vif-EloC, pST39-Vif-EloB-EloC, pST39-Vif-EloB-CBF-β, pST39-Vif-EloC-CBF-β and pST39-Vif-EloB-EloC-CBF-β for expression of proteins or protein complexes in *E*. *coli*. In these plasmids, sequences encoding the following proteins were cloned between the indicated restriction sites: Vif, *Xba*I and *Bam*HI; CBF-β, *Eco*RI and *Hin*dIII; EloC, *Sac*I and *Kpn*I; and EloB, *Bsp*EI and *Mlu*I [63]. The CBF-β gene was acquired by RT-PCR as described below.

VR-EloB/VR-Flag-EloB and VR-EloBΔC34/VR-Flag-EloBΔC34 were generated by subcloning EloB/Flag-EloB and EloBΔC34/Flag-EloBΔC34 into the VR1012 vector at the *Pst*I and *Bam*HI restriction sites. VR-Vif-HA, VR-Vif, VR-VifΔSLQ, VR-Vif-Myc, VR-VifΔSLQ-Myc, VR-EloB-Myc, VR-EloB-HA, VR-EloC-HA and pcDNA3.1-A3G-HA have been previously described [13,27,77]. VR-EBΔ9-17-HA, VR-EBΔI34-HA, VR-EBΔ69-72-HA, VR-EBΔ9-11-HA, VR-EBΔ12-14-HA and VR-EBΔ15-17-HA were derived from VR-EloB-HA via site-directed mutagenesis. VR-P53-HA and the adenovirus E4orf6 expression plasmid were generous gifts from Xiao-Fang Yu (Johns Hopkins University, Baltimore, MD) [73].

The infectious molecular clone pNL4-3ΔVif was obtained from the National Institutes of Health AIDS Research and Reference Reagents Program (NIH-ARRRP), Division of AIDS, National Institute of Allergy and Infectious Diseases (NIAID) in Germantown, MD.

### Cells, antibodies and transfections

HEK293T (CRL-11,268) cells were purchased from the American Type Culture Collection (ATCC, Manassas, VA, USA). MAGI-CCR5 cells (catalog no. 3522) were obtained through the NIH-ARRRP. The cells were maintained in Dulbecco’s modified Eagle’s medium (DMEM) supplemented with 10% fetal bovine serum (FBS) at 37°C and 5% CO_2_. The following antibodies were used in this study: anti-HA mouse monoclonal antibody (mAb) (Covance, Emeryville, CA), anti-Myc mouse mAb (Millipore, Billerica, MA), anti-Flag mouse mAb (Sigma–Aldrich, St. Louis, MO, USA), anti-P53 mAb (Santa Cruz Biotechnology, Santa Cruz, CA), anti-human ribosomal P antigen (Immunovision, Springdale, AR), anti-tubulin mouse mAb (Covance), anti-Cul5 rabbit polyclonal antibody (pAb) (Santa Cruz Biotechnology), anti-ElonginB rabbit pAb (Abcam, Cambridge, MA), anti-ElonginC (BD Transduction Lab, San Jose, CA), anti-CBF-β (Santa Cruz Biotechnology), anti-Vif antibody (NIH-ARRRP, catalog no. 2221) and a monoclonal anti-HIV-1 capsid antibody generated from an HIV-1 p24 hybridoma (NIH-ARRRP) to detect Pr55Gag and CAp24. Plasmid transfections into 293 T cells were performed with Lipofectamine 2000 according to the standard protocol provided by the manufacturer (Invitrogen, Carlsbad, CA).

### RNA interference (RNAi) and qRT-PCR

The following double-stranded siRNAs specific to human EloB were purchased from Shanghai GenePharma Co., Ltd (Shanghai, China): EloB-siRNA1 (siEB1), GGGAAGCAGUGCCAAUGAATT; EloB-siRNA2 (siEB2), UGACCAACUCUUGGAUGAUTT; EloB-siRNA3 (siEB3), GACGAUGGCCAAGAGCAGATT. The AllStars Negative Control siRNA was obtained from Qiagen (siNC, catalog no. 1,027,284, Hilden, Germany). Hiperfect transfection reagent (Qiagen) was used for transfection of 293 T cells with EloB siRNAs at a final concentration of 20 nM according to the manufacturer’s recommendations (Qiagen). Protein expression was monitored by immunoblotting 3 days after transfection. Efficiency of siRNA silencing of EloB was evaluated both by evaluating relative gene expression using qRT-PCR and protein expression by Western blot. Total cellular RNA was isolated from the siRNA-transfected 293 T cells using TRIzol (Invitrogen) and used as the template in cDNA synthesis with the Promega Reverse Transcription System (Promega, Madison, WI). qRT-PCR analysis for *EloB* and *β*-*actin* was performed using the 2× QuantiTect™ SYBR Green PCR Master Mix (Qiagen), C1000™ Thermal Cycler (BioRad CFX 96™ Real-Time System, Hercules, CA) and PCR primers for *EloB* (forward, CGAACTGAAGCGCATCGTC; reverse, TCCAAGAGTTGGTCATCCTTGT) and *β*-*actin* (forward, AGCGGGAAATCGTGC; reverse, CAGGGTACATGGTGGTGC).

The cDNA acquired above was also used as the template for CBF-β amplification using the following primers based on primers described before [33]: forward (5-’ GGAATTCCATATGCCGCGCGTCGTG-3’) and reverse (5’-CGCGGATCCCTAGGGT CTTGTTGTCTTCTTGC-3’). *Nde*I and *Bam*HI restriction sites (in bold) were included in the primers to facilitate cloning into pET3aTr.

### Co-immunoprecipitation assay

At 48 h post-transfection, 293 T cells were harvested, washed with cold PBS and solubilized in lysis buffer (50 mM Tris, pH 7.5, 150 mM NaCl, 1% Triton X-100) plus Complete protease inhibitor cocktail tablets (Roche, Mannheim, Germany) at 4°C for 1 h. The cell lysates were clarified by 30 min of centrifugation at 10,000 × *g* and then incubated with anti-HA Ab-conjugated agarose beads (Roche) at 4°C for 3 h. Alternatively, the pre-clarified lysates were incubated with mouse anti-Myc (Millipore) for 3 h, and Protein G-agarose was then added for a 3-h incubation at 4°C. Subsequently, the samples were washed three times with washing buffer (20 mM Tris, pH = 7.5, 100 mM NaCl and 0.05% Tween 20), boiled in SDS sample buffer and then analyzed by SDS-PAGE and immunoblotting.

### Virus purification and viral infectivity (MAGI) assay

Virus particles were harvested 48 h after transfection, and culture supernatants obtained were clarified by centrifugation at 3000 rpm for 10 min and then filtered through a 0.22-μm pore size membrane. Virions were pelleted through a 20% sucrose cushion by ultracentrifugation at 26,000 rpm for 2 h at 4°C in a Beckman SW40 rotor. For immunoblotting, virions were disrupted in radioimmunoprecipitation assay (RIPA) buffer. Virus infectivity was examined by the MAGI assay as described previously [13,78]. Two days after infection, blue cells and syncytia were counted to determine viral infectivity. Virus input was normalized by the level of p24.

### CHX Vif stability assay

293 T cells were grown to confluence in 6-well culture dishes. At 36 h post-transfection, cells were treated with CHX (Sigma–Aldrich) at the final concentration 100 μg/ml for various time periods, lysed and analyzed by Western blotting.

### Solubility analysis

Individual proteins and protein complexes were expressed in *E*. *coli* BL21 (DE3) cells containing appropriate pST39 constructs. Cells were grown in Luria-Bertani (LB) medium with 50 mg/L ampicillin at 37°C until an optical density (OD) of about 0.8 was reached. The proteins were over-expressed at 37°C for 7 h by induction with isopropyl-D-thiogalactopyranoside (IPTG, 0.5 mM final concentration). Harvested cells were resuspended in PBS (pH 7.4), homogenized by sonication and then clarified by centrifugation at 16,000 × *g* for 1.5 h. For solubility analysis, the supernatant was carefully removed, and the pellet was washed with PBS twice and then resuspended in a volume equal to the original volume of the supernatant.

### Western blotting

Cells and viruses were harvested at 48 h post-transfection and solubilized with RIPA buffer. Samples were boiled in SDS sample buffer for 20 min, subjected to Tricine-SDS–PAGE and then transferred onto nitrocellulose membranes (Whatman, Kent, UK). Secondary antibodies used in this study were alkaline phosphatase-conjugated affinipure goat anti-rabbit IgG, goat anti-mouse IgG and goat anti-human IgG (Jackson Immunoresearch Laboratories, West Grove, PA). Immunoreactions were detected with 5-bromo-4-chloro-3-indolylphosphate (BCIP) and nitro blue tetrazolium chloride (NBT) solutions.

## Abbreviations

CBF-β/CBF: Beta core binding factor beta; Cul5: Cullin5; EB/EloB: ElonginB; EBΔC34: ElonginB mutant with a deletion of the 34 amino acids in the C-terminal tail; EC/EloC: ElonginC; HIV-1: Human immunodeficiency virus type 1; mAb: Monoclonal antibody; pAb: Polyclonal antibody; qRT-PCR: Quantitative real-time reverse transcription polymerase chain reaction; RNAi: RNA interference; siEB: EloB-specific siRNAs; siRNA: Small interfering RNA; siNC: Negative Control siRNA; UbL: Ubiquitin-like; Vif: Virus Infectivity Factor; VifΔSLQ: The Vif SLQ → AAA mutant; VifΔPPL: Vif PPL → AAA mutant.

## Competing interests

The authors declare that they have no competing interests.

## Authors’ contributions

XDW, XHY and WK conceived and designed the experiments and wrote the paper. XDW, XYW, TZ and HW performed the experiments. XDW, HHZ and MYL analyzed the data. JWW, DLL, JYZ, XL, JXW and BY helped design the study and provided reagents. All authors read and approved the final manuscript.
